# Schizencephaly revisited

**DOI:** 10.1007/s00234-018-2056-7

**Published:** 2018-07-19

**Authors:** Paul D. Griffiths

**Affiliations:** Academic Unit of Radiology, University of Sheffield, Floor C, Glossop Road, Sheffield, England S10 2JF UK

**Keywords:** MR imaging, Schizencephaly, Pediatric, Fetus

## Abstract

**Purpose:**

In this paper, I will report the range of appearances of schizencephaly in children and fetuses by reviewing a 10-year experience from a single centre and detail classification systems for the different forms of schizencephaly. This will lead to re-assessment of possible aetiological and mechanistic causes of schizencephaly.

**Methods:**

All cases of pediatric and fetal schizencephaly were located on the local database between 2007 and 2016 inclusive. The studies were reviewed for the presence, location and type of schizencephaly, as well as the state of the (cavum) septum pellucidum, the location of the fornices and the presence of other brain abnormalities.

**Results:**

The review included 21 children and 11 fetuses with schizencephaly. Schizencephaly (type 1) was found in 9% of children but no fetuses, schizencephaly (type 2) was present in 67% of the pediatric cases and in 45% of fetuses, whilst schizencephaly (type 3) was present in approximately 24% of children and 55% of fetuses. Other brain abnormalities were found in 67% of children and 55% of fetuses.

**Conclusion:**

I have proposed a new system for classifying schizencephaly that takes into account all definitions of the abnormality in the literature. Using that approach, I have described the appearances and associations of pediatric and fetal cases of schizencephaly from a single centre. Review of the current literature appears to favour an acquired destructive aetiology for most cases of schizencephaly, and I have proposed a mechanism to explain the cortical formation abnormalities found consistently in and around areas of schizencephaly.

## Introduction

Schizencephaly is a rare congenital abnormality of the brain that has a prevalence of approximately 1.5 per 100,000 live-born babies as estimated from a review of four million births in California [1]. Howe and colleagues reported a combined live birth/stillbirth rate of 1.48 per 100,000 from a population of over 2.5 million in the UK and less than half of those cases were detected antenatally [2]. In spite of recent interest in a range of genetic abnormalities that are associated with schizencephaly [3–5], the majority of cases of schizencephaly are sporadic and non-familial, and in most cases, an aetiological cause is not found [6].

Magnetic resonance (MR) imaging is central to the diagnosis of children with congenital brain abnormalities, and in utero MR (iuMR) imaging is now used for antenatal detection of brain abnormalities, including schizencephaly. The primary purpose of this paper is to assess the range of appearances of schizencephaly in children and fetuses by review of a 10-year experience from a single centre. I will also detail the classification systems used to describe different forms of schizencephaly and re-consider the possible aetiological/mechanistic causes.

## Methods

### Definition

**Fig. 1:**
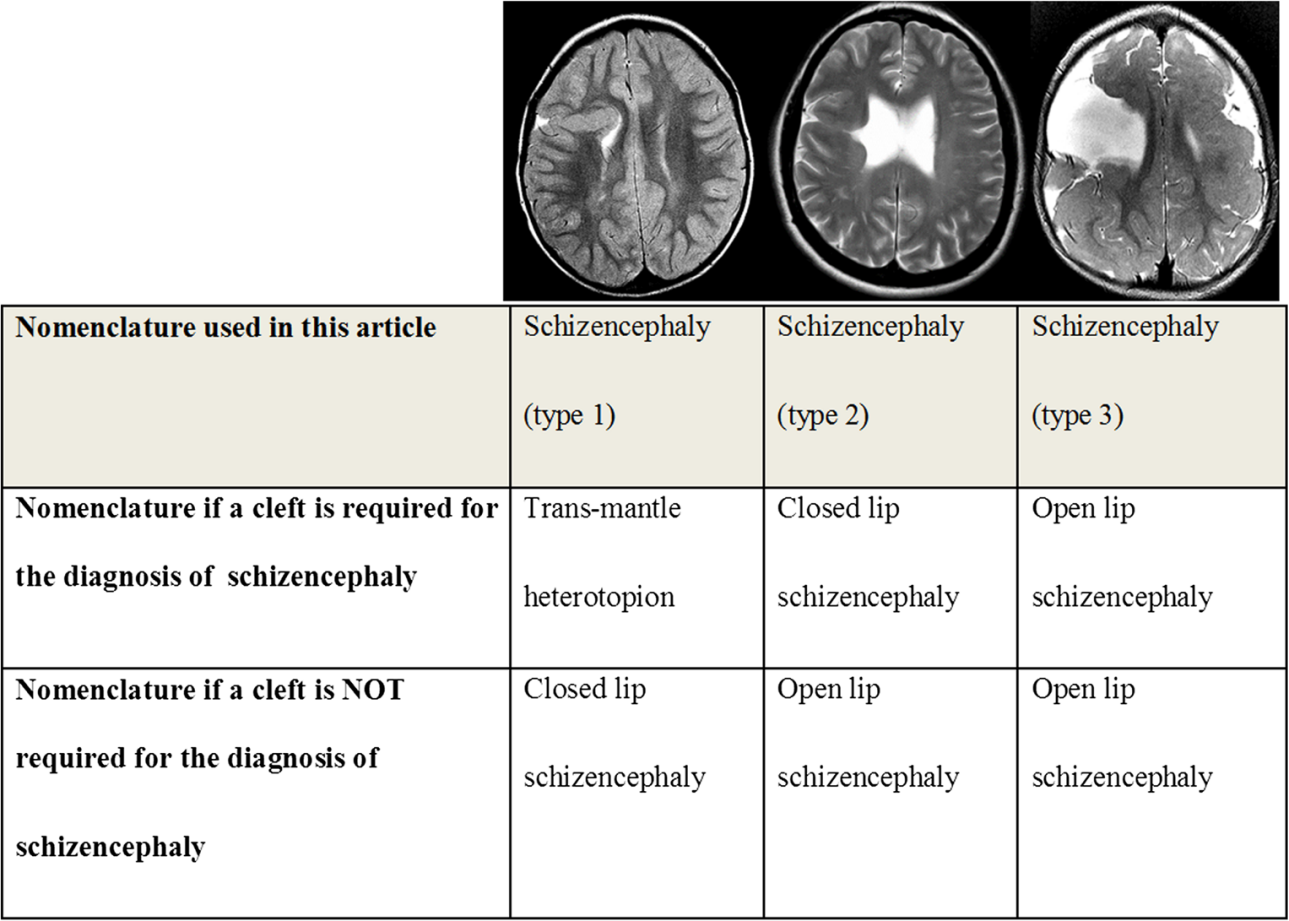
Classification of the three different types of schizencephaly used in this paper compared with other nomenclature systems with pictorial examples (see text for details)

There is a range of opinion about the definition of schizencephaly; specifically, some authorities consider a CSF-containing cleft running from the pial to the ependymal surfaces to be the sine qua non of schizencephaly [7–9]. In contrast, the definition of schizencephaly recently put forward by Naidich et al. includes a trans-mantle column of dysplastic grey matter extending from the ependyma to the pia without a CSF cleft [10]. Those authors reference supportive evidence from the publications of Yakovlev and Wadsworth [11, 12] and specimens housed in the Yakovlev-Haalem collection at the Armed Forces Institute of Pathology, Washington, DC. The differences in nomenclature used by the two groups are summarised in Fig. 1 along with a unifying system that will be used in this article (below and Fig. 1).

- *Schizencephaly (type 1)*—Trans-mantle column of abnormal grey matter but no evidence of a CSF-containing cleft on MR imaging
- *Schizencephaly (type 2)*—CSF-containing cleft present, abutting lining lips of abnormal grey matter opposed
- *Schizencephaly (type 3)*—CSF-containing cleft present, non-abutting lining lips of abnormal grey matter

**Fig. 2:**
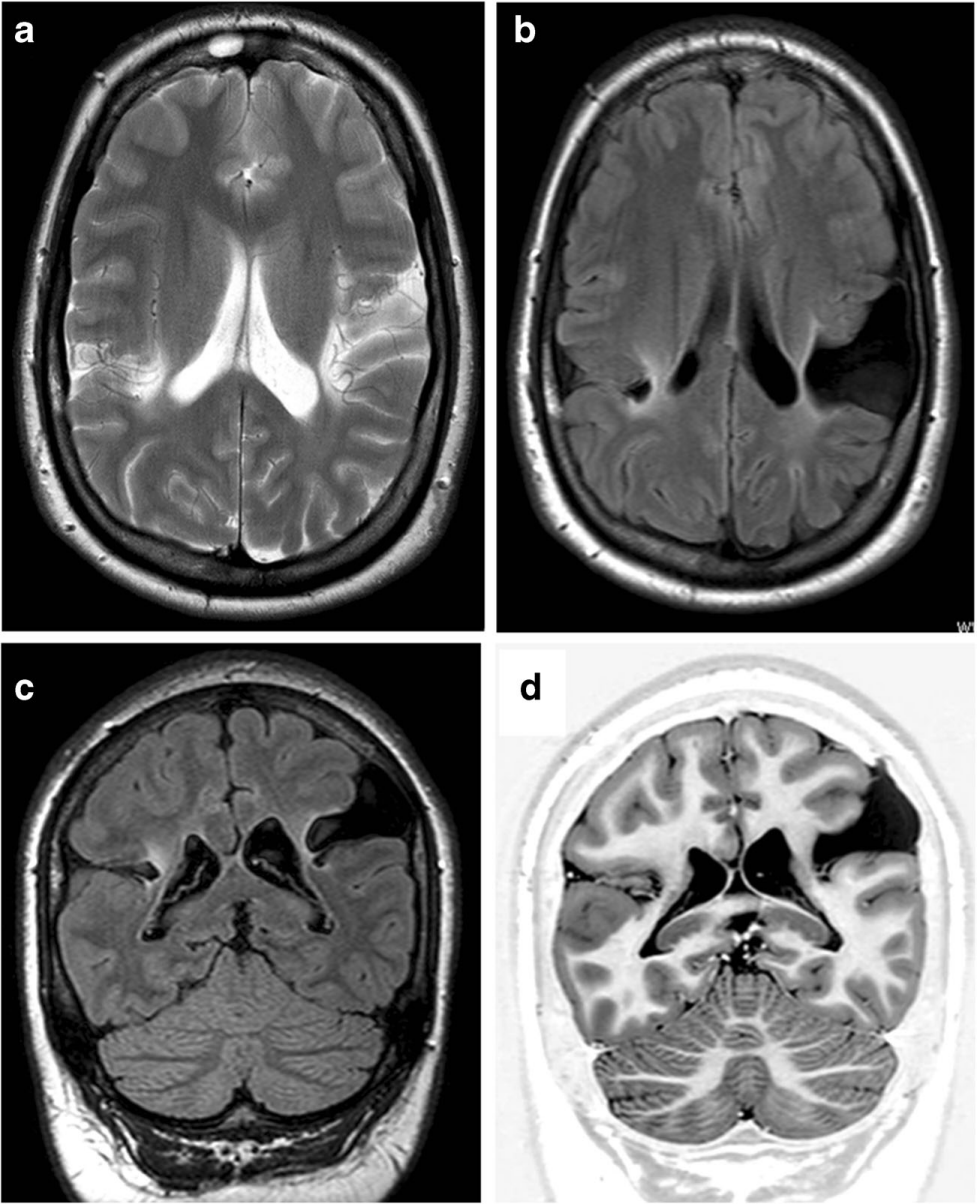
A child with porencephaly. MR imaging of an 8-year-old child with spastic quadriplegic cerebral palsy recognised in the first year of life. Axial T2-weighted (**a**), axial FLAIR (**b**), coronal FLAIR (**c**) and coronal inversion recovery (**d**) show bilateral clefts involving the paracentral lobules, which extend from the outer surface of the brain but do not quite reach the ventricular margin. Some of the white matter next to the clefts is gliotic and there is no evidence of normal, or abnormal, grey matter lining the clefts. These features indicate porencephaly rather than schizencephaly

If two types of schizencephaly are present in the same individual, it is described in this paper by the numerically higher category; e.g. if a child has an open and a closed lip cleft, the individual will be classified as having schizencephaly (type 3). Figure 2 shows MR imaging from a child with porencephaly, the major differential diagnosis of schizencephaly and distinguished by the lack of abnormal grey matter lining the cleft.

### Caseload

The cases of schizencephaly described in this paper are all from the Academic Unit of Radiology, University of Sheffield, and the MR imaging was performed between 2007 and 2016 inclusive. The local database was searched for all cases of schizencephaly reported on MR studies during that 10-year period in children (aged 16 years or younger) and fetuses following in utero MR (iuMR) studies. All of the studies involving children and some of the iuMR studies were performed for clinical purposes, and the other iuMR studies were performed as part of research studies. The pregnant women recruited into research studies all provided written informed consent for the iuMR imaging if it was performed as a research study and, although they were not paid for their involvement, travel expenses were provided for the woman and a companion. Relevant review was sought, and approval obtained, from the Institutional Clinical Effectiveness Unit and Research Department in order to allow those cases performed for clinical purposes to be reported in this paper.

### MR imaging technique

MR imaging for children was performed on either 1.5-T (Infinion, Philips Medical Systems, Cleveland, OH, or HDx General Electric Healthcare, Milwaukee, WI) or 3.0-T MR scanners (Achieva, Philips Medical Systems, Best, Netherlands), whereas iuMR imaging was performed only on the 1.5-T scanners. The children in the study had the following MR sequences performed as a minimum: axial and coronal FSE T2-weighted, T1 volume imaging (1.5 mm partition thickness maximum), axial FLAIR and axial gradient echo T2* imaging. The iuMR studies consisted of ssFSE imaging in the three orthogonal planes (5 mm thickness maximum), ultrafast axial T1 and axial diffusion-weighted imaging as a minimum. All fetuses imaged after 2013 had steady-state 3D volume imaging [13].

### Analysis

All of the studies were reported clinically by a number of radiologists, but all of the studies were reviewed for the purposes of this paper by the author, an experienced pediatric neuroradiologist (over 25 years of pediatric neuroradiology and over 18 years of iuMR imaging experience). The presence, location and type of schizencephaly were recorded for each case as well as the condition of the (cavum) septum pellucidum, the location of the fornices and the presence of other brain abnormalities.

## Results

### Pediatric cases

**Fig. 3:**
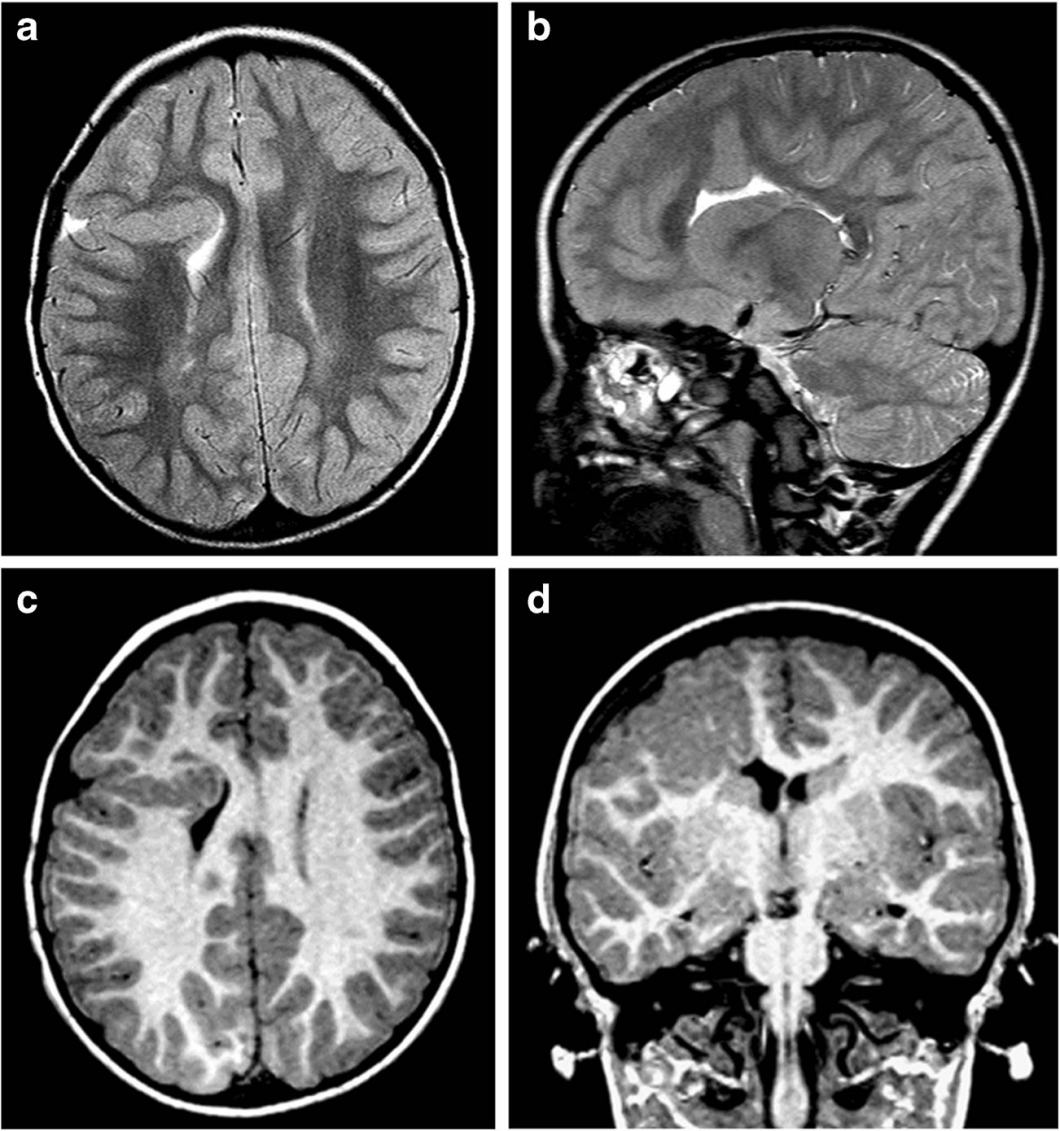
Unilateral schizencephaly (type 1). MR images of a 4-year-old child with focal epilepsy. Axial T2-weighted (**a**), right parasagittal T2-weighted (**b**), axial (**c**) and coronal reconstructions from T1 volume imaging show abnormal grey matter extending from ventricular to out surface of the brain, centred on the right middle frontal gyrus. No CSF cleft is visible, hence the classification as schizencephaly (type 1). The septum pellucidum is present and the course of the fornices is normal. No other brain abnormalities are present

**Fig. 4:**
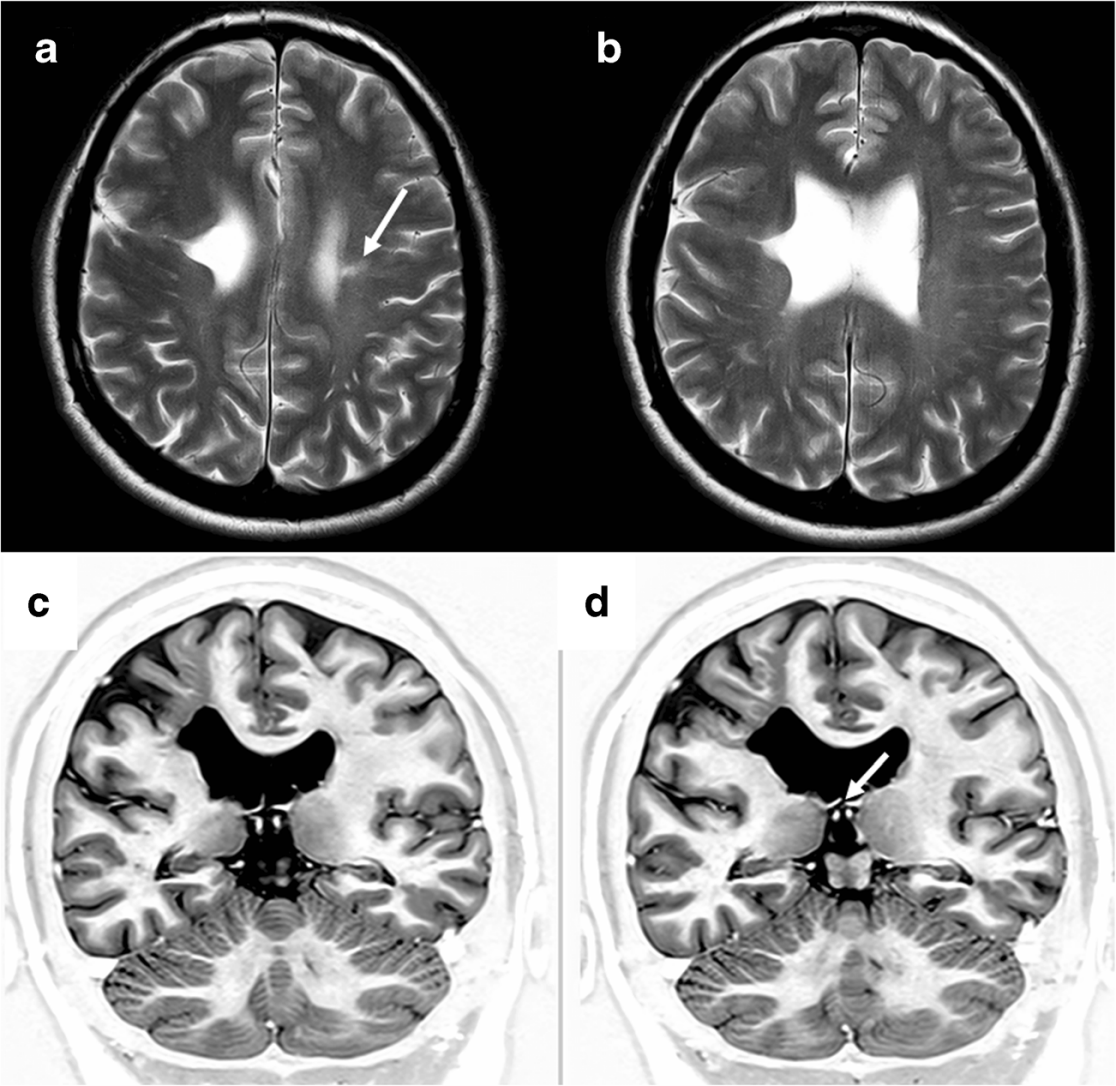
Unilateral schizencephaly (type 2). MR images of a 4-year-old child with focal epilepsy and developmental delay. Axial T2-weighted (**a**, **b**) and coronal inversion recovery (**c**, **d**) images show a CSF cleft with closely opposed borders lined with polymicrogyria. There is also a small area of gliosis in the white matter of the contralateral paracentral lobule (arrowed on **a**). The septum pellucidum is absent and the fornices lie abnormally low (arrowed on **d**). No other brain abnormalities were present

**Fig. 5:**
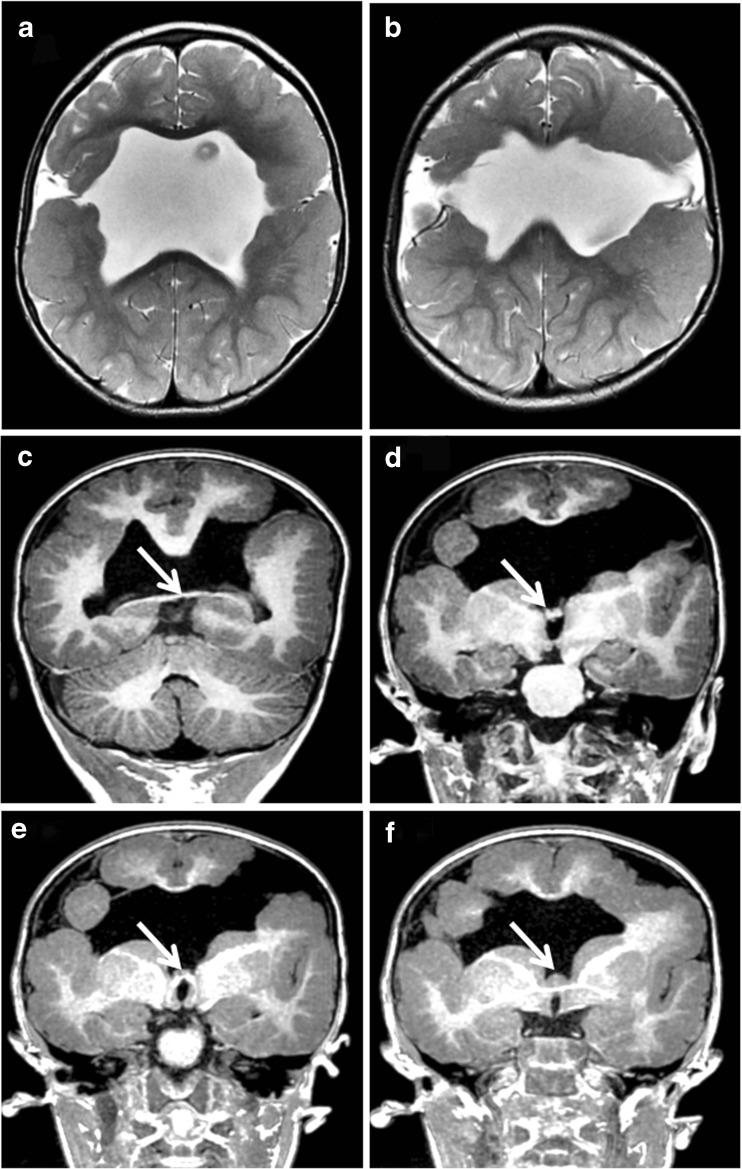
Bilateral schizencephaly (type 3). MR images of a 2-year-old child with global developmental delay. Axial T2-weighted (**a**, **b**) and coronal reconstructions from T1-weighted volume data (**c**–**f**) show bilateral CSF clefts with non-opposed borders lined with polymicrogyria. The septum pellucidum is absent and the fornices lie abnormally low (arrowed on **c**–**f**). Extensive cortical formation abnormalities were present in both hemispheres

**Fig. 6:**
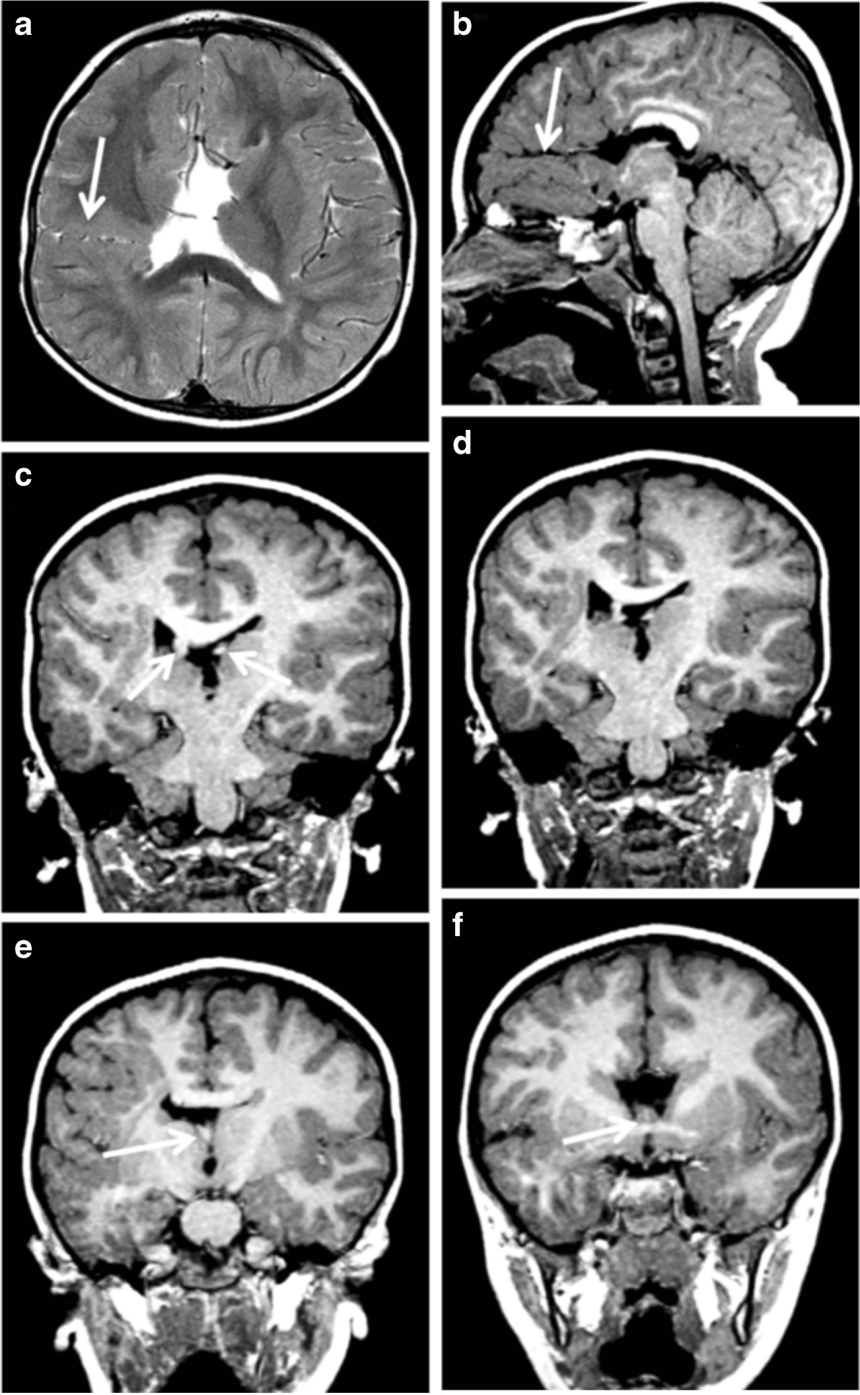
Two regions of schizencephaly (type 2) in the same hemisphere. MR images from a 2-year-old child with hemiparietic (left side of body) cerebral palsy. Axial T2-weighted image shows schizencephaly (type 2) related to the right paracentral lobule (arrowed on **a**) whilst the right parasagittal image from a T1 volume acquisition shows a further area of schizencephaly (type 2) in the inferior frontal gyrus (arrowed on **b**). The anterior part of the corpus callosum is absent and there is a low position of cerebellar tonsils. Coronal reconstructions from the T1 volume data from posterior to anterior (**c**–**f**) show an aberrant course of the fornices and distorted, thickened septum pellucidum (arrowed on **c**, **e** and **f**)

Clinical and MR imaging summaries of 21 children with schizencephaly are shown in Table 1: 14/21 (67%) pediatric cases in this series had schizencephaly (type 2), 5/21 (24%) had schizencephaly (type 3), whilst schizencephaly (type 1) was present in only 2/21 (9%). Examples are shown in Figs. 3, 4 and 5. Just under half had bilateral schizencephaly and 3/21 children had more than one cleft in a cerebral hemisphere (Fig. 6).

**Table 1:** Clinical and radiological summaries of 21 children with schizencephaly

Case	Age at MR	Neurodevelopmental information	Schizencephaly: Number Laterality Symmetry	Schizencephaly: Location/type	Other brain abnormalities	Fornix location	Septum pellucidum on MR	SOD suspected clinically
P1*	7 years	Microcephaly, quadriparetic cerebral palsy, global developmental delay	2 Bilateral Symmetric	Right: paracentral lobule/type 2 (closed lip) Left: paracentral lobule/type 2 (closed lip)	Extensive bilateral PMG	Normal	Present	No
P2*	7 months	Microcephaly, in utero growth restriction, quadriparetic cerebral palsy	2 Bilateral Symmetric	Right: paracentral lobule/type 2 (closed lip) Left: paracentral lobule/type 2 (closed lip)	Extensive bilateral PMG	Normal	Present	No
P3	2 years	Global developmental delay, visual impairment	2 Bilateral Symmetric	Right: paracentral lobule/type 3 (open lip) Left: paracentral lobule/type 3 (open lip)	Extensive bilateral PMG	Superior aspect of the 3rd ventricle	Absent	Yes
P4	14 months	Hemiparetic cerebral palsy (right)	1 Unilateral	Left: middle frontal gyrus/type 2 (closed lip)	Contralateral posterior peri-sylvian PMG	Superior aspect of the 3rd ventricle	Absent	Yes
P5	2 years	Global developmental delay, epilepsy	3 Bilateral Non-symmetric	Right: a. superior frontal gyrus/type 2 (closed lip) Right: b. superior temporal gyrus/type 1 (no cleft) Left: superior frontal gyrus/type 1 (no cleft)	ACC, type 2 cysts, extensive nodular heterotopia	Aberrant	Absent	No
P6	3 years	Epilepsy	1 Unilateral	Right: superior temporal gyrus/type 2 (closed lip)	No	Normal	Present	No
P7	13 years	Microcephaly, global developmental delay, epilepsy	2 Bilateral Symmetric	Right: extensive frontal/type 3 (open lip) Left: extensive frontal/type 3 (open lip)	No	Normal	Disrupted	No
P8	8 days	Hypoglycaemia, hyponatraemia	1 Unilateral	Left: paracentral lobule/type 2 (closed lip)	No	Superior aspect of the 3rd ventricle	Absent	Yes
P9	2 months	Unilateral ptosis (right)	1 Unilateral	Right: paracentral lobule/type 2 (closed lip)	Contralateral heterotopia	Normal	Disrupted	Yes
P10	10 months	Epilepsy	1 Unilateral	Right: paracentral lobule/type 2 (closed lip)	Mirror contralateral PMG	Superior aspect of the 3rd ventricle	Absent	Yes
P11	7 months	Microcephaly, global developmental delay, epilepsy	3 Bilateral Non-symmetric	Right: paracentral lobule/type 3 (open lip) Left: a. paracentral lobule/type 2 (closed lip) Left: b. inferior frontal gyrus (pars opercularis)/type 3 (open lip)	Extensive bilateral PMG Hypogenesis CC, absent splenium	Superior aspect of the 3rd ventricle	Absent	Yes
P12	3 years	Diplegic cerebral palsy	2 Bilateral Symmetric	Right: paracentral lobule/type 2 (closed lip) Left: paracentral lobule/type 2 (closed lip)	No	Normal	Present	No
P13	11 months	Hemiparetic cerebral palsy (left)	1 Unilateral	Right: paracentral lobule/type 2 (closed lip)	Mirror contralateral PMG	Superior aspect of the 3rd ventricle	Absent	No
P14	2 years	Global developmental delay, epilepsy	2 Bilateral Symmetric	Right: middle frontal gyrus/type 2 (closed lip) Left: middle frontal gyrus/type 2 (closed lip)	Extensive bilateral PMG	Superior aspect of the 3rd ventricle	Absent	No
P15	4 years	Epilepsy	1 Unilateral	Right: middle frontal gyrus/type 1 (no cleft)	No	Normal	Present	No
P16	13 months	Hemiparetic cerebral palsy (right)	2 Bilateral Non-symmetric	Right: middle frontal gyrus/type 2 (closed lip) Left: extensive frontal/parietal lobes/type 3 (open lip)	Extensive bilateral PMG Body of CC absent? disrupted	Superior aspect of the 3rd ventricle	Absent	No
P17	4 years	Global developmental delay, epilepsy	1 Unilateral	Right: paracentral lobule/type 2 (closed lip)	No	Superior aspect of the 3rd ventricle	Absent	No
P18	9 years	Global developmental delay, epilepsy	1 Unilateral	Right: superior frontal gyrus/type 2 (closed lip)	No	Superior aspect of the 3rd ventricle	Absent	Yes
P19	2 years	Hemiparetic cerebral palsy (left)	2 Unilateral	Right: a. paracentral lobule/type 2 (closed lip) Right: b. inferior frontal gyrus (pars orbitalis)/type 2 (closed lip)	Hypogenesis CC (genu/anterior body absent) Chiari 1 malformation	Aberrant	Absent	Yes
P20	12 months	Global developmental delay, epilepsy, macrocephaly	1 Unilateral	Left: supramarginal gyrus/type 1 (no cleft)	Hypogenesis CC (posterior body/splenium absent)	Aberrant	Disrupted	No
P21	5 years	Global developmental delay, epilepsy	2 Bilateral Symmetric	Right: inferior frontal gyrus (pars opercularis)/type 3 (open lip) Left: inferior frontal gyrus (pars opercularis)/type 3 (open lip)	Agenesis CC, bilateral PMG, fused nucleus accumbens septi	Superior aspect of the 3rd ventricle	Absent	Yes

**Fig. 7:**
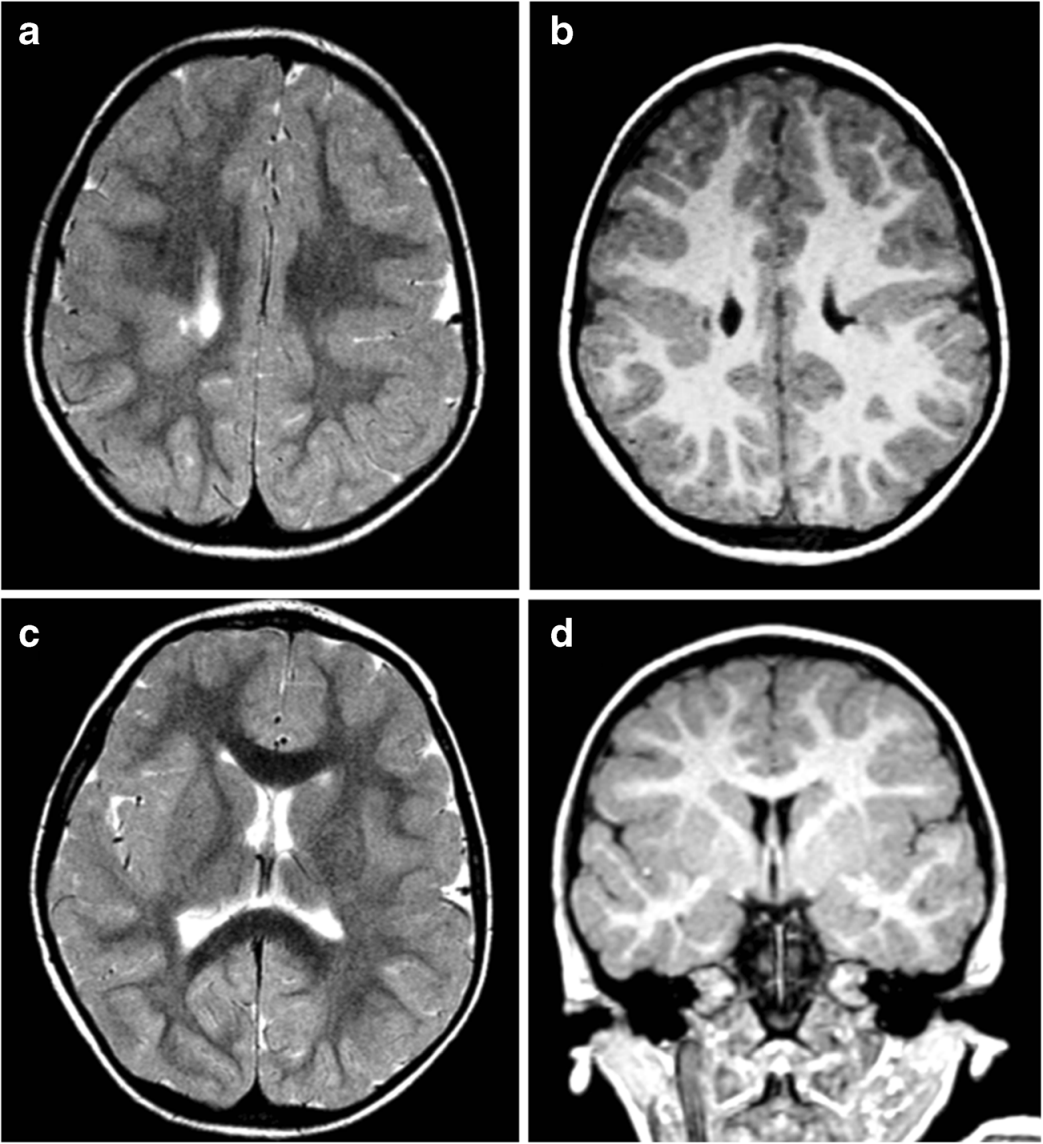
**a**–**d** Bilateral schizencephaly (type 2) with an intact septum pellucidum, normal course of the fornices, and no other brain abnormalities in a 3-year-old child with diplegic cerebral palsy

**Fig. 8:**
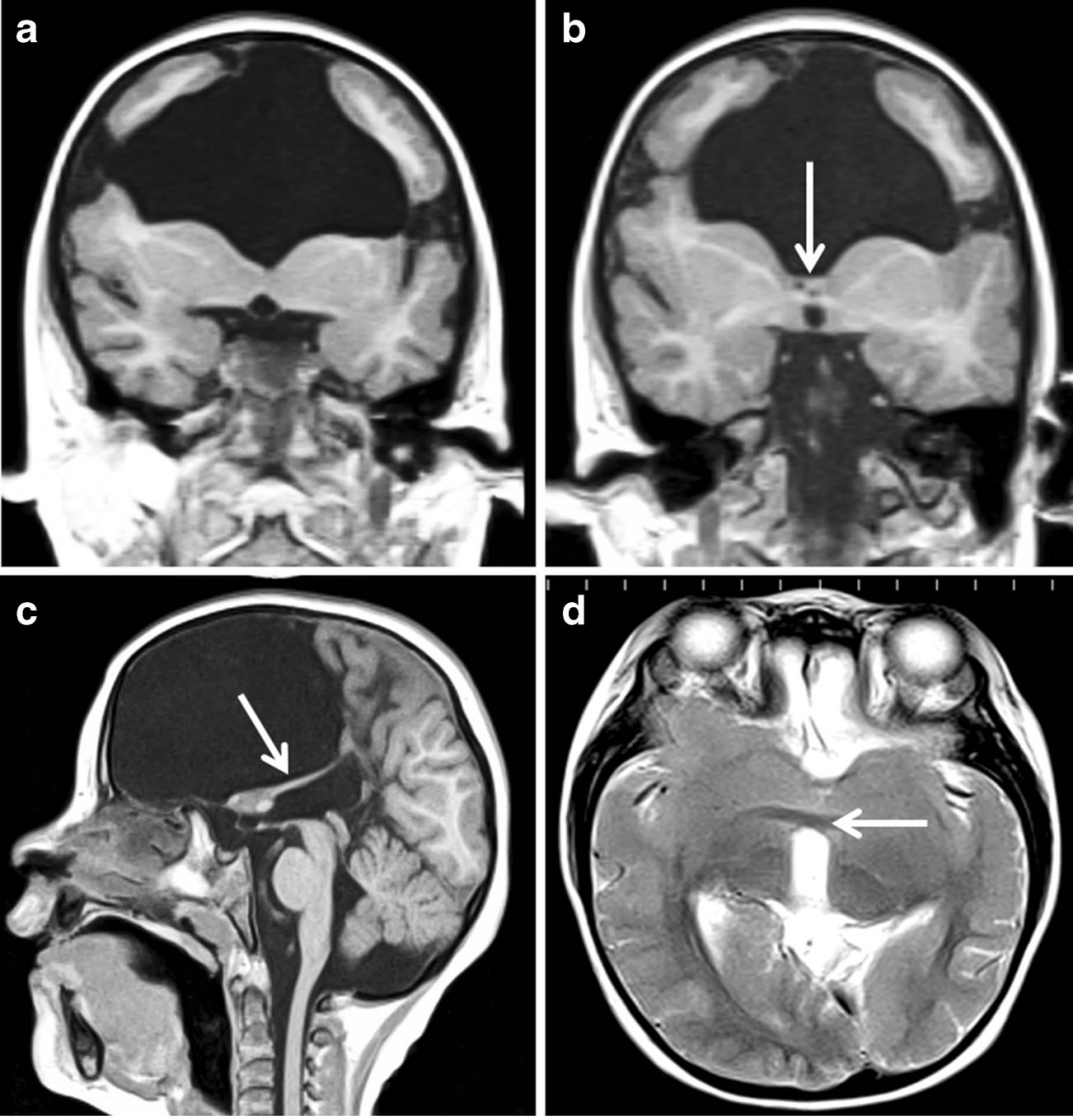
Bilateral schizencephaly (type 3) with multiple other brain abnormalities. MR imaging of a 5-year-old child with global developmental delay and epilepsy. Coronal reconstructions from T1 volume datasets (**a**, **b**) show bilateral schizencephaly (type 3) involving the inferior frontal gyri. Bilateral cortical formation abnormalities are present in the superior portions of the frontal lobes. The corpus callosum and septum pellucidum are absent and the fornices have an abnormal low position (arrowed on **b** and **c**). Axial T2-weighted image shows abnormal fusion of the nucleus accumbens septi and basal forebrain across the midline (arrowed on **d**) which possibly represents a form of septo-preoptic holoprosencephaly

The septum pellucidum was present and intact in 5/21 (24%) of the pediatric cases (Fig. 7) and was absent in 13/21 (62%) of children and disrupted in 3/21 (14%) cases. The course of the fornices was normal [14] in all children with intact septum pellucidum, passing close to the splenium of the corpus callosum, suspended from the inferior margin of the septum pellucidum and showing the expected relationship with the anterior commissure at its termination (Figs. 3 and 7). The course of the fornices was also normal in two thirds of the children with disrupted septum pellucidum. The fornices were present but sited abnormally low [14, 15] in 11/13 children without a septum pellucidum (superior portion of the third ventricle—Figs. 4, 5 and 8). Three children (two with no septum pellucidum and one with a disrupted septum pellucidum) had an aberrant course of the fornices (Fig. 6) all of whom had failed commissuration, which probably accounted for the aberrant course [15].

**Fig. 9:**
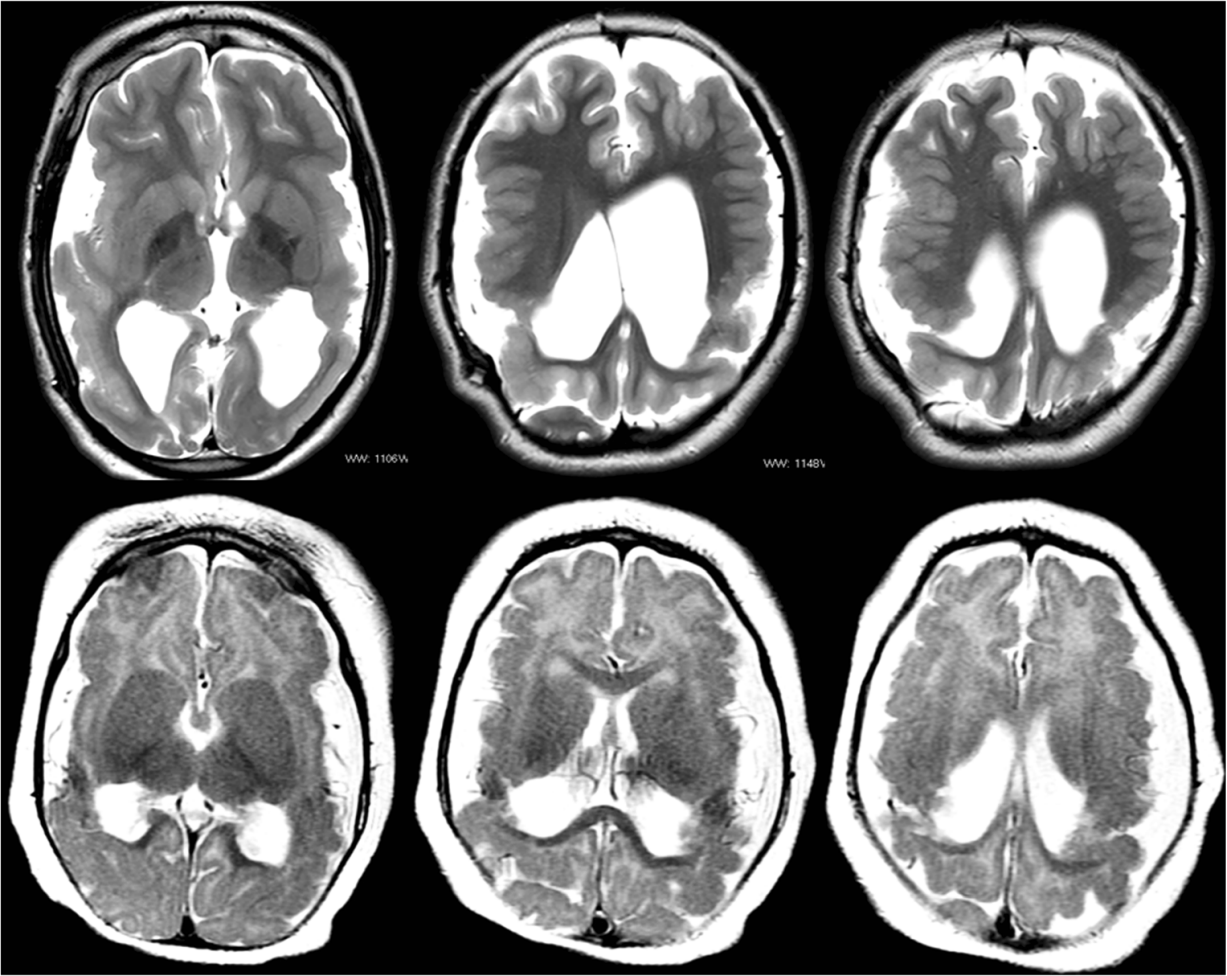
MR images of a 7-year-old child (upper pane of the images) with bilateral schizencephaly (type 2) and known EMX2 genetic mutation and MR images of a 7-month-old child with a strong family history of schizencephaly but no genetic testing at the time of the MR study (lower pane of the images). The two cases show similar imaging features as discussed in the text

Other brain abnormalities (not including the septum pellucidum/fornix or polymicrogyria localised to the vicinity of the cleft) were found in two thirds (14/21) of the children. Cortical formation abnormalities were the commonest, found in 12/21 (57%) pediatric cases of which 10 were polymicrogyria. Failed commissuration was present in 5/21 (24%) pediatric cases—two cases of agenesis (Fig. 8) and three cases of hypogenesis of the corpus callosum. The two cases of known or suspected genetic causes are shown in Fig. 9.

### Fetal cases

**Fig. 10:**
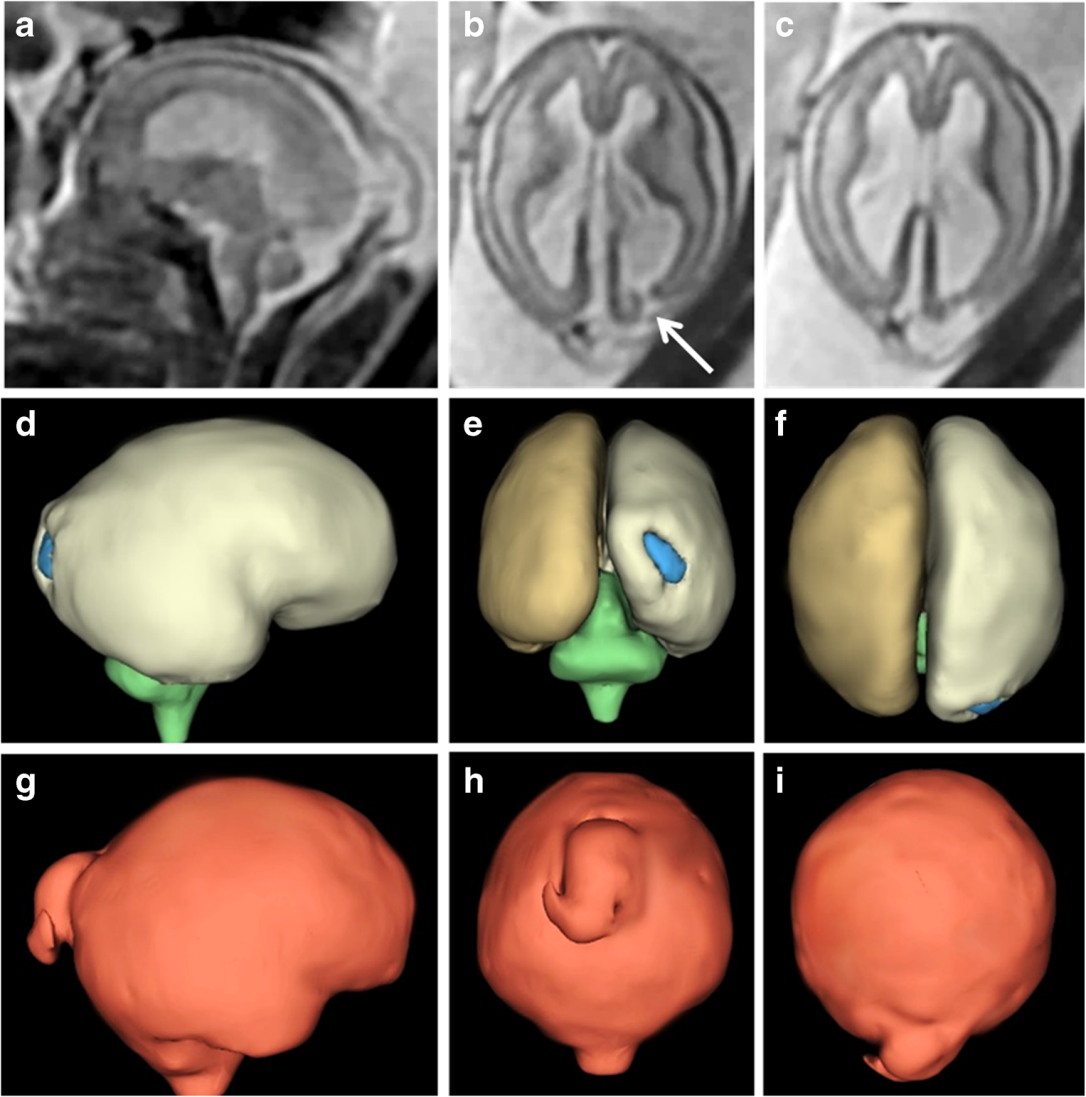
Unilateral schizencephaly (type 2) in a 21gw fetus. Sagittal (**a**) and axial (**b**, **c**) ultrafast T2-weighted images show a cleft in the right occipital lobe with opposed deep portions (arrowed on **b**). Subependymal heterotopia was also present (not shown). Those images are reversed to be consistent with the constructed model of the brain (**d**—right lateral, **e**—posterior and **f**—superior) which show the superficial part of the cleft is quite wide. There is a midline meningocoele posteriorly which is well shown on the models of the external CSF spaces (**g**–**i**)

**Fig. 11:**
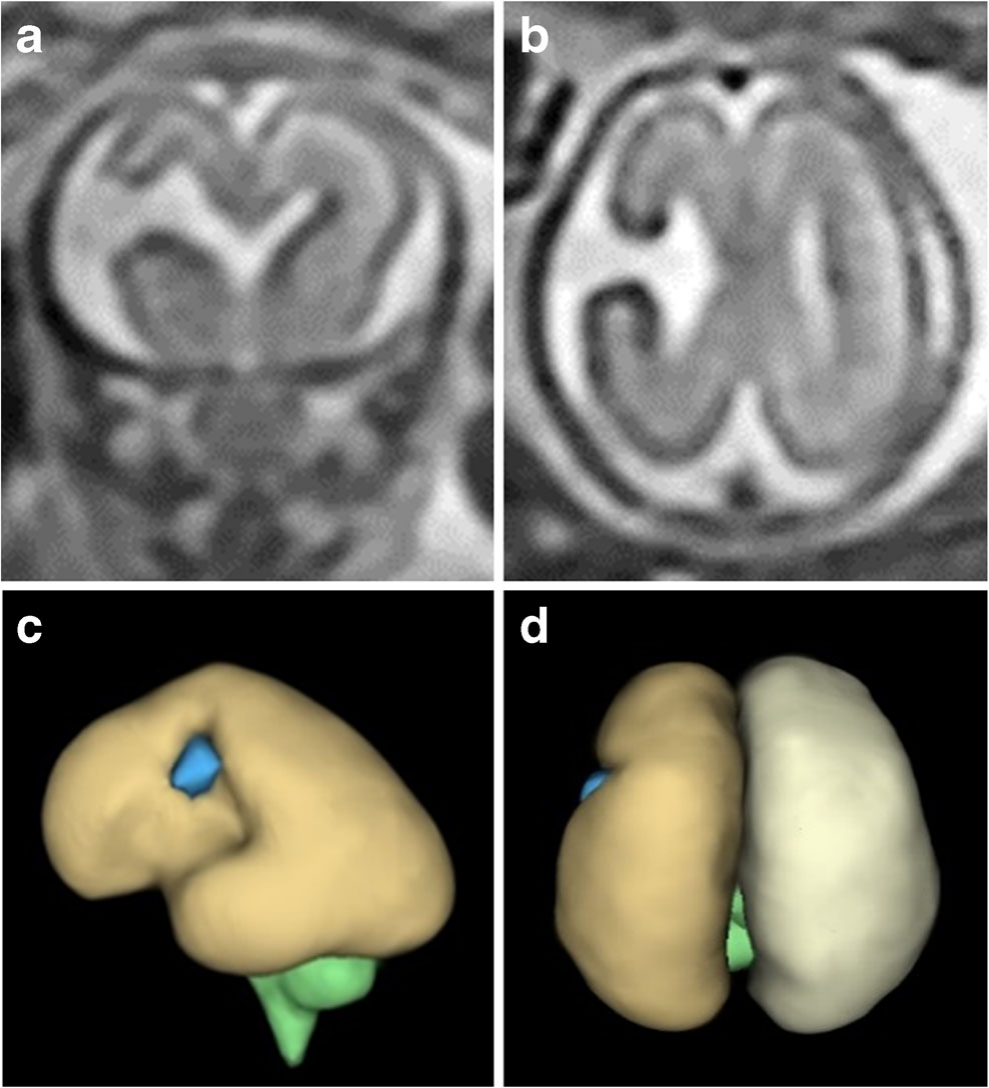
Unilateral schizencephaly (type 3) in a 21gw fetus. Coronal (**a**) and axial (**b**) ultrafast T2-weighted images show a widely spaced cleft in the right paracentral lobule. Those images are reversed to be consistent with the constructed models (**c**—left lateral and **d**—superior) of the brain constructed from a 3D steady-state acquisition. The septum pellucidum is absent but no other brain abnormality was shown

The iuMR imaging summaries of 11 fetuses with schizencephaly are shown in Table 2, and data comparing the findings in children and fetuses are presented in Table 3. Schizencephaly (type 2) was present in 5/11 (45%) fetuses (Fig. 10) and schizencephaly (type 3) in 6/11 (55%) fetuses (Fig. 11). No fetus had schizencephaly (type 1). Fetal head size ≤ 10th centile were found in 6/11 cases, and in 4/11, the head size was < 3rd centile, all of whom had schizencephaly (type 3). Unilateral schizencephaly was present in 7/11 (64%) and bilateral involvement in 4/11 (36%). No fetus had multiple areas of schizencephaly in the same cerebral hemisphere.

**Table 2:** Imaging summaries of 11 fetuses with schizencephaly

Case	Gestational age at MR	Head size (bi-parietal diameter)	Schizencephaly: Number Laterality Symmetry	Schizencephaly: Location/type	Cavum septum pellucidum	Fornix location	Other brain abnormalities
F1	22gw	3–10th centile	1 Unilateral	Right: inferior frontal gyrus/type 2 (closed lip)	Absent	Superior aspect of the 3rd ventricle	No
F2	21gw	10–50th centile	1 Unilateral	Right: occipital lobe/type 2 (closed lip)	Present	Normal	1. Cephalocele 2. Heterotopia
F3	33gw	< 3rd centile	2 Bilateral Asymmetric	Right: extensive frontal lobe/type 3 (open lip) Left: paracentral lobule/type 3 (open lip)	Disrupted	Normal	No
F4	21gw	10–50th centile	1 Unilateral	Left: paracentral lobule/type 3 (open lip)	Absent	Superior aspect of the 3rd ventricle	No
F5	28gw	90th centile	1 Unilateral	Right: parietal lobe/type 2 (closed lip)	Present	Normal	Polymicrogyria of adjacent brain
F6	25gw	10th centile	1 Unilateral	Left: paracentral lobule/type 2 (closed lip)	Absent	Superior aspect of the 3rd ventricle	Extensive bilateral polymicrogyria
F7	27gw	< 3rd centile	1 Unilateral	Left: middle and inferior frontal gyrus and paracentral/type 3 (open lip)	Absent	Superior aspect of the 3rd ventricle	Contralateral polymicrogyria
F8	26gw	< 3rd centile	2 Bilateral Symmetric	Right: parietal lobe/type 3 (open lip) Left: parietal lobe/type 3 (open lip)	Present	Normal	Microencephaly, encephalomalacia
F9	21gw	3–10th centile	2 Bilateral Asymmetric	Right: paracentral lobule/type 2 (closed lip) Left: extensive frontal/type 3 (open lip)	Present	Normal	No
F10	26gw	< 3rd centile	2 Bilateral symmetric	Right: paracentral lobule/type 3 (open lip) Left: paracentral lobule/type 3 (open lip)	Absent	Superior aspect of the 3rd ventricle	No
F11	31gw	50–90th centile	1 Unilateral	Right: middle frontal gyrus/type 2 (closed lip)	Absent	Superior aspect of the 3rd ventricle	Contralateral focal megalencephaly

**Table 3:** Summary of MR imaging findings compared between the pediatric and fetal cases of schizencephaly

	Schizencephaly (type 1)	Schizencephaly (type 2)	Schizencephaly (type 3)	Unilateral	Bilateral	Present	Absent	Disrupted	Yes	No
Pediatric cases (*n* = 21)	2 (9%)	14 (67%)	5 (24%)	11 (52%)	10 (48%)	5 (24%)	12 (57%)	4 (19%)	17 (81%)	4 (19%)
Fetal cases (*n* = 11)	0 (0%)	5 (45%)	6 (55%)	7 (64%)	4 (36%)	4 (36%)	6 (55%)	1 (9%)	6 (55%)	5 (45%)

The septum pellucidum was present and intact in 4/11 (36%) fetuses (Fig. 10) and absent in 6/11 (55%) fetuses (Fig. 11), and disrupted remnants of the septum pellucidum were shown in 1/11 (9%). The path of the fornices was normal in all fetuses when the septum pellucidum was present or disrupted, whilst the fornices were abnormally low (superior portion of the third ventricle) in all cases of absent septum pellucidum.

Other brain abnormalities (not including the septum pellucidum/fornix) were found in 6/11 (55%) fetuses—five had cortical formation abnormalities and the other fetus had focal encephalomalacia. Failed commissuration was not seen in any of these fetuses.

## Discussion

Twenty-one consecutive cases of pediatric schizencephaly and 11 cases of fetal schizencephaly are reported in this article in order to illustrate the range of appearances that can be expected in clinical practice. For most radiologists, schizencephaly is a term used to describe congenital (present at birth) brain abnormality in which the defining lesion is a full-thickness CSF cleft lined by abnormal grey matter (polymicrogyria and/or heterotopia). The grey matter lining distinguishes it from porencephaly, which results from a destructive lesion in the fetal brain white matter, lined with variable degrees of gliosis, (Fig. 2), some of which can be minimal. CSF-containing clefts can be missed on MR imaging, particularly if imaging in the three orthogonal planes is not used, and 3D T1 volume imaging with thin partitions is ideal for this role once myelination is mature. In spite of using best quality imaging, a cleft can still be overlooked and a dimple or ‘nipple’ on the ventricular surface may be the only indicator of trans-mantle abnormality on MR imaging. I have noted another definition of schizencepahly that does not require a cleft to be present [10–12], and recent editions of Barkovich and Raybaud’s textbook acknowledge ‘…transmantle heterotopia may be an extreme closed lip schizencephaly’ [8]. Those differences in definition of schizencephaly can cause confusion when attempting to sub-classify types of schizencephaly, hence my attempt to produce the unifying system shown in Fig. 1.

Allowing for the fact that many authors consider the schizencephaly (type 1) cases in this paper to be trans-mantle heterotopion, there are inevitable differences in the anatomical descriptions of schizencephaly found in textbooks and previous papers when compared with the current cohort. It is noteworthy that there were no fetuses with schizencephaly (type 1) in this cohort, which may be due to the limitations of iuMR imaging in terms of anatomical resolution. Barkovich reports bilateral schizencephaly in 40–50% of pediatric cases [8], which is comparable with the findings reported here (just less than 50% in children and 36% in fetuses), but there were substantial differences in the category of schizencephaly. ‘Closed lip’ schizencephaly is found in only 15–20% of children according to the textbook in comparison with schizencephaly (type 2) being found in 67% of children and 50% of fetuses in this study. The frontal and parietal preponderance reported by Barkovich (about 75%) was re-demonstrated and strengthened here (94% of all schizencephalies in both the pediatric and fetal cohorts). Textbook estimates of the absence of the septum pellucidum in children with schizencephaly are around 70% (optic nerve hypoplasia in approximately 30%) and were similar in this report (absent or disrupted septum pellucidum in 76% of pediatric cases and 64% of fetuses). The tendency for absent septum pellucidum to be associated with ‘open lip’ (schizencephaly (type 3) reported previously was also shown in our cases. Other brain abnormalities (not including the septum pellucidum/fornix) were found in approximately two thirds of the children which is comparable to previous reports from pediatric populations.

There are limited reports of fetal schizencephaly using iuMR imaging—for example, small case series [16] and case reports [17–19] with the largest case series to date by Nabavizadeh et al. [20] who reported 10 fetuses with schizencephaly on iuMR imaging and confirmed on post-natal imaging including eight bilateral cases. One interesting finding in that study was a morphological change in the type of cleft between the pre-natal and post-natal imaging as 47% of the ‘open-lip’ schizencephalies detected in utero had become ‘closed lip’ on post-natal imaging. Another important point was how frequently polymicrogyria was missed on iuMR imaging in that study, and this may account for the low rate of other brain abnormalities shown on iuMR in the present study (55% compared with 81% in children).

**Fig. 12:**
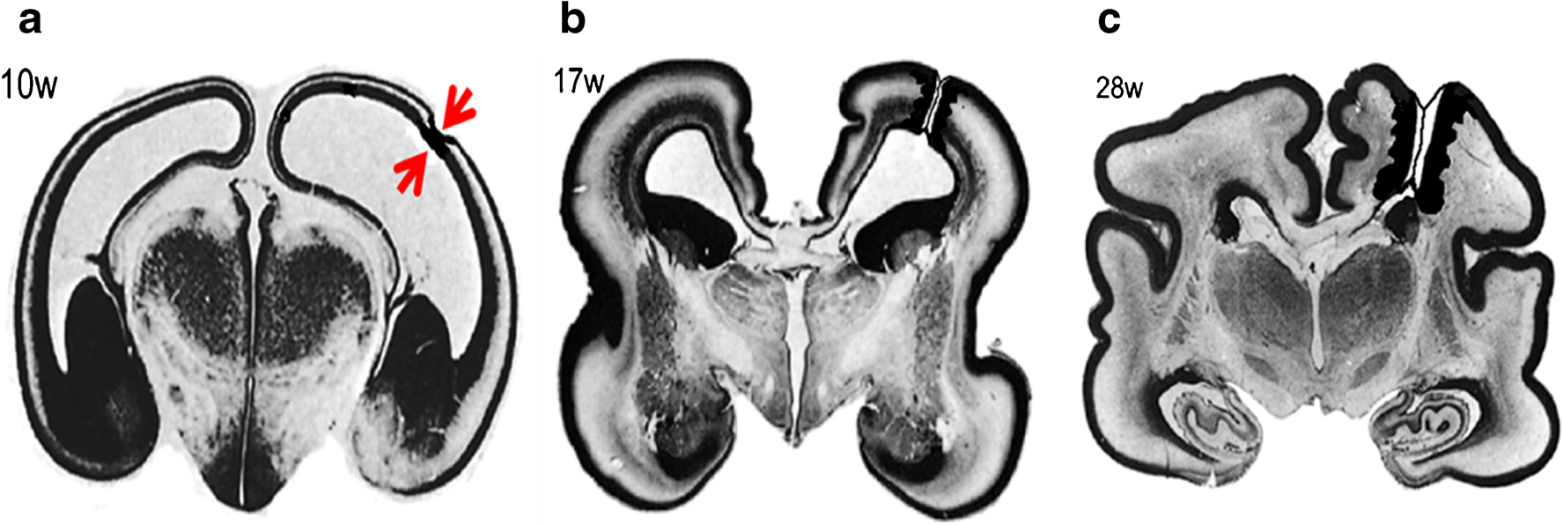
Representation of the ependymal/pial pinning theory of schizencephaly. Coronal histology sections of the fetal brain at 10gw (**a**), 17gw (**b**) and 28gw (**c**) illustrate early pinning of the ependymal and pia (arrowed on **a**) with the development of an ependymal/pial seam. These sections have been reproduced after alteration with permission [23]

One mechanistic explanation of the aetiology of schizencephaly arises from the work of Yakovlev and Wadsworth in 1946 [11, 12]. In their opinion, schizencephaly is due to ‘failure of growth and differentiation of a circumscribed part of the cerebral wall’ as quoted in Greenfield’s neuropathology [21]. As such, it has its origins in the first trimester, specifically the first 2 months post-conception. Naidich [22] elaborated and refined the theory by proposing pinning of the ependymal and pia, which are anatomically very close in the first trimester (Fig. 12). The focal non-separation leads to an ependymal/pial seam that lies between the lips of the abnormal developing cleft. The ependymal/pial pinning theory may explain some features of schizencephaly (type 2) lesions where the lips are closely opposed, but intuitively, it seems difficult to explain the widely spaced lips and volume loss frequently present in schizencephaly (type 3). The proponents of this idea merely suggest that larger areas of ependyma and pia fail to separate in those cases.

**Fig. 13:**
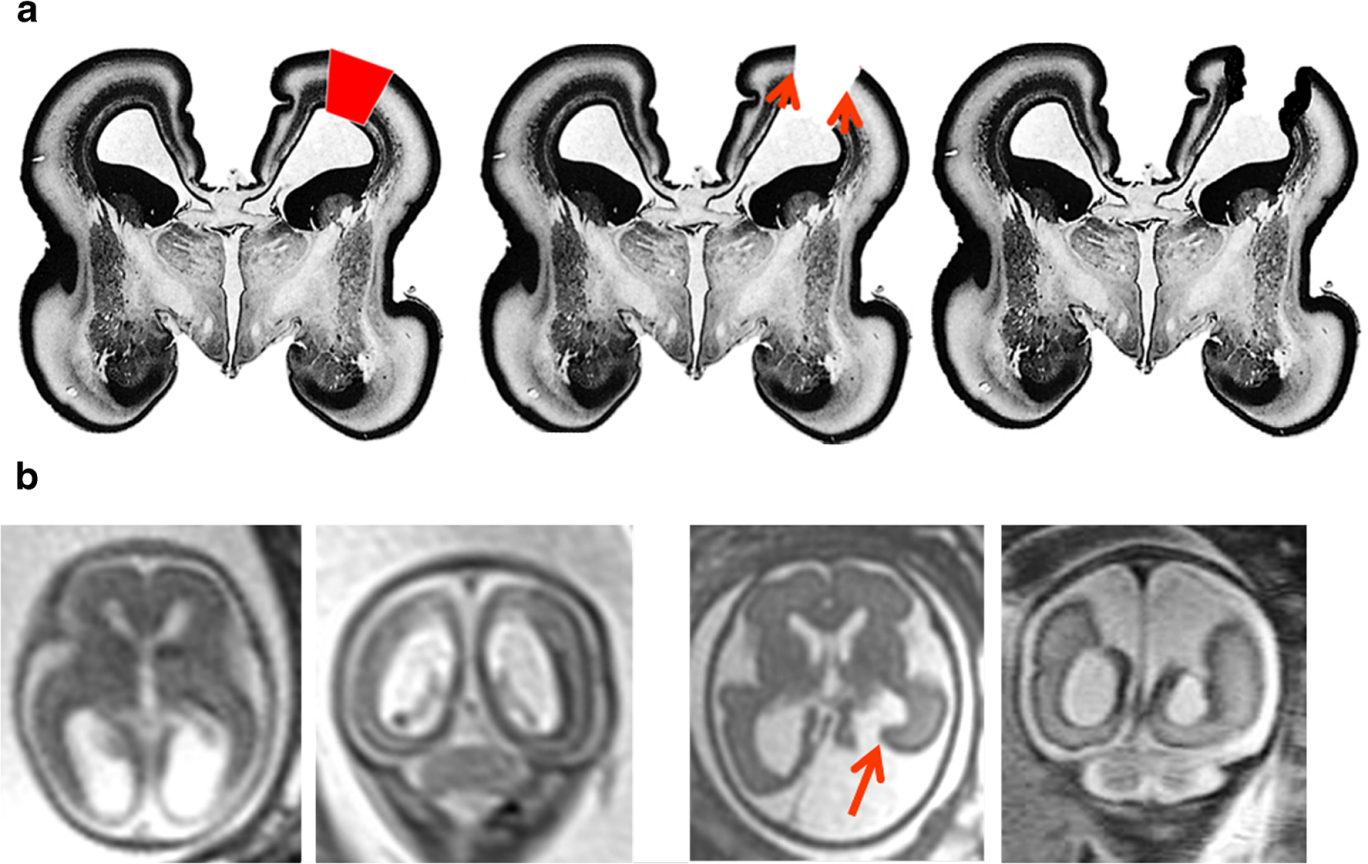
Representation of the destructive theory of the formation schizencephaly. Coronal histology sections of the fetal brain at 17gw (**a**) have been reproduced after alteration with permission [24]. The red quadrilateral on figure **a** represents a focal, full-thickness injury to the cortical mantle and resorption of the damaged brain. Neuro-glial migration is still possible at this stage and will form the regions of polymicrogyria in the borders of the schizencephalic cleft. **b** iuMR images from a fetus imaged on two occasions because of the history of monochorionic pregnancy and selective termination of one twin. Imaging at 21gw shows high signal and loss of volume in the otherwise normally formed posterior portions of both hemispheres. Repeat iuMR at 27gw shows the development of schizencephaly (type 3) in the left posterior hemisphere. This case is not from our cohort but shown courtesy of Professor M. Kilby, University of Birmingham, and has been described elsewhere [25]

There is opposition to the pinning theory in recent neuropathology texts, and Harding and Copp, writing in Greenfield’s neuropathology, state ‘… interpretation of the apposed walls of the cleft and the bridging membrane over the defect is no longer convincing, although for obscure reasons it lingers in the radiological literature. There is now ample clinical, morphological and experimental evidence favouring a destructive origin for these lesions’ [21]. Those authors go on to say that further evidence against the pinning theory is the consistent presence of abnormal cortex lining the clefts, which points to an aetiological cause in the second trimester: ‘…timing is suggested by the coincident polymicrogyria to be between the fourth and sixth months’. Most textbooks now consider sporadic schizencephaly (the huge majority of cases) to be the result of a destructive lesion(s): Chen, for example, states that the primary aetiology is due to ‘in utero vascular insufficiency’ [9]. The data emerging from iuMR imaging studies also favour destructive aetiologies in most cases. Circumstantial evidence comes from the work of Nabavizadeh et al. [20] who found that 7/10 fetuses with schizencephaly had hemorrhage and/or hemosiderin deposition in the cleft or in the cerebral ventricles on iuMR imaging. The finding reported in the current paper that schizencephaly in the fetus is accompanied by a small head size in 6/11 fetuses (below the 10th centile) and in 4/11 the head size was below the 3rd centile is relevant because the most rational explanation for the high risk of microcephaly is brain destruction. It is interesting to note that the four fetuses with the head size below the 3rd centile all had schizencephaly (type 3), i.e. cases in which greater volume of brain injury might be predicted. Direct evidence comes from the fetus illustrated in Fig. 13, which is not from the cohort as presented but has been reported previously as web extra material [25]. That case shows the evolution of schizencephaly in a second trimester fetus with a normally formed but injured cerebral mantle. That fetus was studied because it was the result of a monochorionic pregnancy complicated by the death of the co-twin, a known risk factor for schizencephaly [9, 21].

Consistent with many previous descriptions, the septum pellucidum was absent in many cases of schizencephaly, although the reason for the association is not known with certainty. In some of the pediatric and fetal cases reported here, remnants of the septum pellucidum were visualised, suggesting that the structure had formed but was damaged, perhaps by raised intraventricular pressure arising from hydrocephalus secondary to intraventricular blood. It is not possible to say if this mechanism accounts for the case in which no portion of the septum pellucidum was seen.

I explained the need to distinguish between schizencephaly and porencephaly earlier, but from the discussion above, it seems likely that destructive brain parenchymal lesions are the most likely cause for most cases of schizencephaly as well as porencephaly. Why would one fetus develop schizencephaly and another develop porencephaly? It is attractive to propose that the gestational age at which the brain injury occurs is the deciding factor. If the insult occurs in the ‘4–6-month’ time period proposed by Harding and Copp [21], further neuroglial migration is possible and likely after the damaging event. That ‘late migration’ neurons could extend as far as the margins of the injured brain but are not likely to organise properly and result in polymicrogyria (Fig. 13). In contrast, a brain injury occurring after 6 months is most likely to produce an abnormality where the damaged brain is resorbed, and because no further neuroglial migration is possible, the margins are not lined by normal (or abnormal) grey matter. The exact appearances of injuries in that time period will depend on the ability of the brain to produce a gliotic reaction, porencephaly resulting if there is no gliosis (less mature fetuses) or encephalomalacia if there is gliosis (more mature fetuses) [8].

**Fig. 14:**
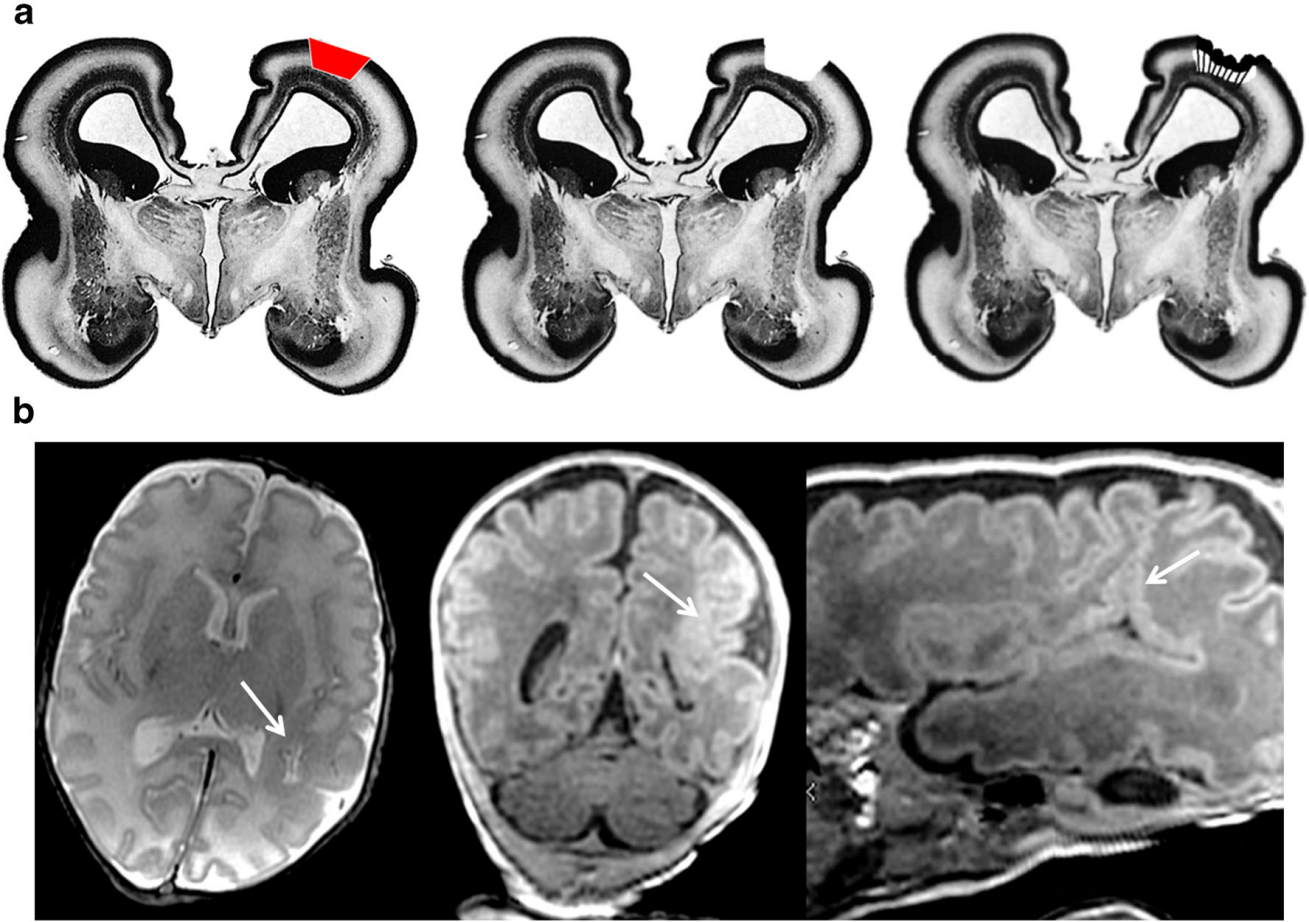
Representation of the destructive theory of the formation of ‘reparative’ polymicrogyria. Coronal histology sections of the fetal brain at 17gw (**a**) have been reproduced after alteration with permission [24]. The red quadrilateral on **a** represents a focal, superficial injury to the cortical mantle, and resorption of the damaged brain is shown. Late neuro-glial migration in this case will produce polymicrogyria on the cortical surface. **b** iuMR images from a 9-week child which resulted from a monochorionic pregnancy complicated by twin-twin transfusion with survival of both twins. Axial T2-weighted and images from a T1 volume study and non-orthogonal along the course of the sylvian fissure show focal polymicrogyria in the abnormal posterior extension of the sylvian fissure (arrowed)

If most cases of schizencephaly are the result of destructive lesions in the second trimester, the damage to the cerebrum must be trans-mantle but it is interesting to consider cases in which a focal brain injury is superficial rather than full thickness. If the injury occurs at the time that further neuroglial migration is possible, the damaged cortical plate may become re-populated by grey matter (Fig. 14). Again, it is not likely to result in normal six-layered cortex but more likely to result in what has been called ‘reparative polymicrogyria’. This is a possible explanation for the association of unilateral schizencephaly (full-thickness vascular injury) and contralateral polymicrogyria (superficial vascular injury) in the same fetus as suggested in a previous work [26].

Other recognised risk factors for the development of schizencephaly include transplacental infections, classically by cytomegalovirus [21] with more recent reports of infection by the Zika virus [27]. Transplacental infection by cytomegalovirus may involve the ventricular layer (germinal matrix) where the future neurons and glia are formed and mature before migration. It is possible that in these circumstances schizencephaly may result from abnormal migration/organisation. Alternatively, cytomegalovirus infection is known to cause arteritis, which potentially could injure previously normal brain and results in schizencephaly by ‘late migration’ mechanism described above. Schizencephaly can also be a feature of many congenital anomaly/mental retardation conditions such as Adam-Oliver, Aicardi, Arima, Delleman, Galloway-Mowat and micro syndromes [21].

Discrete genetic causes of schizencephaly have been difficult to confirm and it is generally considered to be a non-genetic, non-familial ‘sporadic’ abnormality. Early reports linking schizencephaly to mutations in the EMX2 gene have not been borne out [24]. Two cases in our pediatric cohort did have relevant histories: one child had a known mutation of the EMX2 gene and the other had a strong family history of schizencephaly but no known genetic abnormality at the time of the MR study. The MR imaging features of those two children had striking similarities (Fig. 9)—both were microcephalic, both had bilateral schizencephaly (type 2) involving the paracentral lobes and both had extensive bilateral polymicrogyria and intact septum pellucidum with normally situated fornices. The presence of normal septum pellucidums in these cases is particularly noteworthy as the structure was either absent or disrupted in 16/19 (84%) of the other children with schizencephaly.

In conclusion, I have proposed a new system for classifying schizencephaly that takes into account all definitions of the abnormality. Using that approach, I have described the appearances and associations of pediatric and fetal cases of schizencephaly from a single centre. Review of the current literature appears to favour an acquired destructive aetiology for most cases of schizencephaly, and I have proposed a mechanism to explain the cortical formation abnormalities (predominantly polymicrogyria) found consistently in and around areas of schizencephaly.
